# Diagnostic Overshadowing and Pain Insensitivity in a Schizophrenic Patient With Perforated Duodenal Ulcer

**DOI:** 10.7759/cureus.21800

**Published:** 2022-02-01

**Authors:** Akhil Kallur, Eungjae Yoo, Fred Bien-Aime, Hussam Ammar

**Affiliations:** 1 Internal Medicine, MedStar Washington Hospital Center, Washington, USA

**Keywords:** surgical acute abdomen, perforated peptic ulcer, schizophrenia, pain insensitivity, diagnostic overshadowing

## Abstract

Some patients with schizophrenia and psychotic illnesses have reduced pain perception, and others have decreased pain expression. The diagnosis of the acute abdomen can be delayed, and its outcomes can be worse in psychiatric patients than in non-psychiatric patients. We present a case of perforated peptic ulcer (PPU) in a schizophrenic woman and discuss how the phenomenon of pain insensitivity and diagnostic overshadowing-a process in which a person with mental illness receives inadequate treatment due to a misattribution of physical symptoms to their mental illness-nearly contributed to a missed diagnosis.

## Introduction

The stigma attached to mental illness is the main obstacle to the provision of care for people with this disorder. Stigma leads to discrimination in the provision of services for physical illness in those who are mentally ill and to low use of diagnostic procedures when they have physical illness [1]. Psychotic patients with diabetes or coronary artery disease are less likely to be admitted to the hospital for diabetic complications or to receive revascularization procedures during hospitalization than their counterparts with no mental illness [2,3]. Surgical patients with schizophrenia showed significantly higher mortality and postoperative adverse outcome rates [4].

## Case presentation

**Figure 1:**
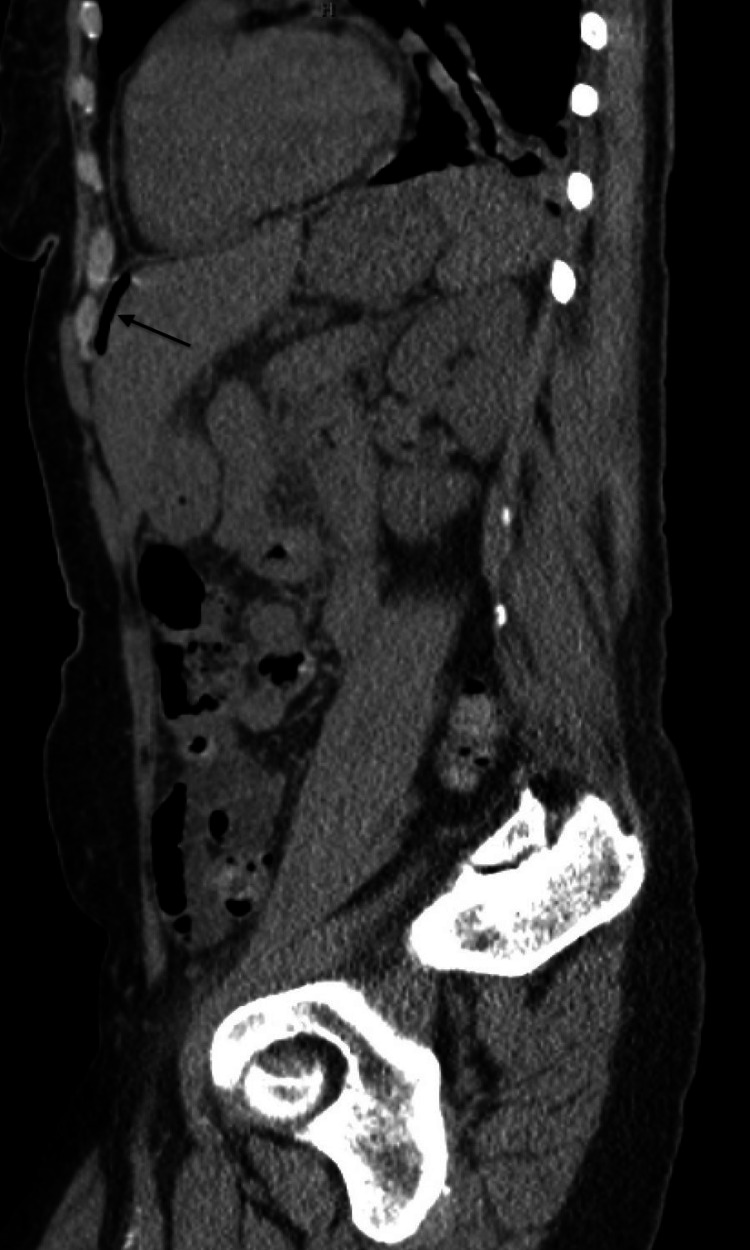
computed tomography sagittal image Pneumoperitoneum (arrows)

**Figure 2:**
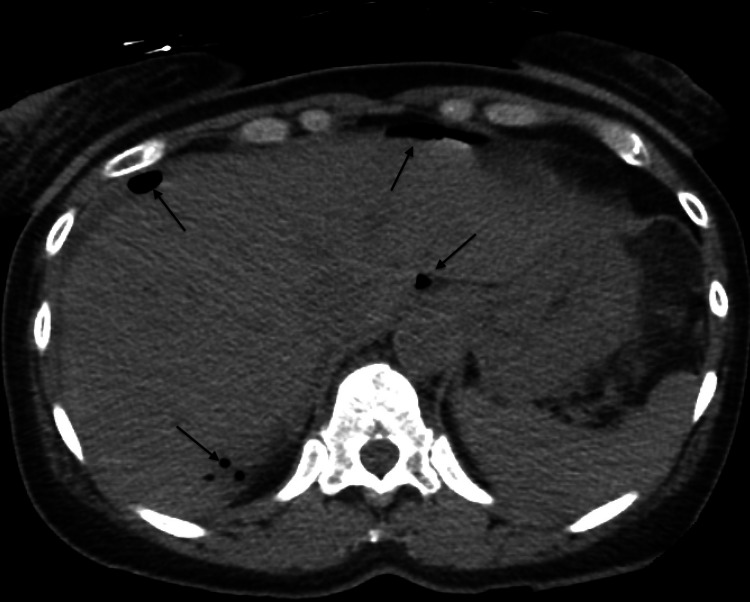
computed tomography axial image Pneumoperitoneum (arrows)

A 62-year-old woman with a history of paranoid schizophrenia was brought to the emergency department (ED) after the emergency medical service was called. She had been found sitting on the floor of her apartment building hallway, complaining of back pain. Her blood pressure (BP) was 111/71 mm Hg, pulse rate was 69 per minute, respiratory rate was 17 per minute, and her temperature was 36.4°C. Physical examination in the ED was unremarkable. She complained of pain throughout her body and nausea and had vomited a few times. She was agitated, and history-taking was challenging. Laboratory tests were significant for a blood urea nitrogen (BUN) of 67 mg/dl and serum creatinine of 2.12 mg/dl (reference range 0.5 to 1.0 mg/dL). Overnight, she received intravenous fluids. BUN and creatinine improved to 47 mg/dl and 1.54 mg/dl, respectively. The next morning, she talked about a person who had been following her constantly and that she could not take it anymore. She was agitated, and the admitting team tried to reassure and calm her down. Prior records indicated that she had refused to take antipsychotics for her paranoid schizophrenia. Her primary care physician mentioned these delusions frequently in his notes and documented history of coronary artery disease and hypertension. The admitting team thought that her worsening delusions might be indirectly contributing to her dehydration and requested an urgent psychiatric consultation. The hospital psychiatric team evaluated the patient and agreed with the diagnosis of paranoid schizophrenia but did not think she needed further psychiatric intervention. She continued to refuse antipsychotics, and the psychiatric team suggested discharge if she was medically stable. Early on the morning of the second day of hospitalization, the medical team was called as the patient requested to be discharged immediately. A urine toxicology screen returned positive for cocaine, the nursing staff documented a good appetite, and the patient took her medicines, including aspirin, atorvastatin, and metoprolol. Upon entering the patient’s room, we found the patient screaming in pain and clinching her abdomen. We tried to reassure her and administered a dose of tramadol 25 mg. Her abdomen was soft, non-distended, and non-tender. An abdominal ultrasound revealed a normal gall bladder with no stones. A blood lipase was only mildly elevated at 60 units/L (normal range 12-53). The patient was calm after she received an intramuscular injection of 1 mg of haloperidol. Intravenous fluids and abdomen computed tomography (CT) were ordered. Early morning on the third day of hospitalization, the patient took off her hospital gown, put on her dress, and requested immediate discharge. The CT revealed pneumoperitoneum and irregularities of the gastric wall and proximal duodenum, suggesting a perforated site (Figures 1, 2).

We walked into the patient’s room. She was sitting in a chair dressed in her clothes; she was calm and did not complain of pain. We explained the scan report; she nodded her head and did not say a word. The patient was taken to the operating room, and a Graham patch repair of the perforated duodenal ulcer was successfully performed. The postoperative course was complicated by a hemoglobin drop that required transfer to the surgical intensive care unit and the need for blood transfusion. The patient was discharged home on postoperative day 12. 

## Discussion

The classic triad of sudden onset of severe epigastric pain, tachycardia, and abdominal rigidity is considered the hallmark of perforated peptic ulcer (PPU) [5]. In an 1843 paper, Edward Crisp first reported 50 cases of peptic perforation and described the clinical aspects of perforation, stating: “The symptoms are so typical, I hardly believe it possible that anyone can fail to make the correct diagnosis” [6]. Atypical presentation of PPU has been more recognized with the introduction of computed tomographic scans in the evaluation of acute abdominal pain. Abrupt onset of severe abdominal pain as described by Crisp was present in only 61.7% of patients with PPU in a retrospective study of 332 patients [5]. The diagnosis of acute surgical conditions in schizophrenic patients is fraught with difficulties since these patients do not always experience pain, which is the cardinal symptom of acute abdomen. In a 1959 study of 79 psychotic patients, 21.4% of patients with PPU and 36.8% of patients with acute appendicitis presented without any complaints of pain [7]. Several case series have reported silent acute abdomen and the absence of peritoneal signs in patients with schizophrenia [8,9]. The mechanism of pain insensitivity in schizophrenic and psychotic patients has not been defined, and it is likely a multifactorial combination of biological, psychological, and sociological elements. Schizophrenic patients frequently shun human contact and may prefer death to the increased human contact necessary for the treatment of illness [8,9]. These patients’ lack of communication and inability to verbalize somatic complaints make them indifferent to pain stimuli. Potential defects in the peripheral and central nervous systems and cognitive loss of the meaning of pain might contribute to pain such as rigidity can also be absent, and the only presenting feature can be a worsening of psychotic symptoms [8,9]. 

Another major cause of diagnostic delay when treating psychotic patients is the phenomenon of diagnostic overshadowing. Diagnostic overshadowing refers to the process by which a person with mental illness receives inadequate treatment because his or her physical symptoms are misattributed to the mental illness [2,3]. Bias or discriminatory attitudes toward psychotic patients might play a role in some cases, as well as physicians’ lack of knowledge about how psychiatric patients communicate and present their physical complaints [2,3].

## Conclusions

Our lack of knowledge regarding the near-silent presentation of acute abdomen in schizophrenic patients contributed to the diagnostic overshadowing of this patient. Clinical providers should avoid diagnostic overshadowing when they treat patients with mental illness. Clinicians should be careful not to fall into the trap of premature closure and making “all in her head” our only diagnosis. Some patients with schizophrenia do not express pain and might have no peritoneal signs but catastrophic intraabdominal pathologies. 
